# Supplementary material for: Effects of Caulerpa taxifolia on Physiological Processes and Gene Expression of Acropora hyacinthus during Thermal Stress
Source: Biology (Basel). 2022 Dec 9;11(12):1792. doi: 10.3390/biology11121792 (PMC9775474; doi:10.3390/biology11121792)
Supplement: Supplementary file 1 [file biology-11-01792-s001.zip › biology-2052935-supplementary.pdf]

Supplementary Material

Table S1: Summary of values of water chemistry data for all treatments.The results are presented as means ± standard deviation.

variable	Coral		Coral exposed to algae		Coral contact with algae	
	27°C	30°C	27°C	30°C	27°C	30°C
Temperature [°C]	27.1±0.1	30±0.2	27.1±0.1	30±0.2	27.1±0.1	30±0.2
PH	8.0±0.03	8.0±0.04	8.0±0.03	8.0	8.0±0.03	8.0
Salinity	32.1±0.4	32.1±0.4	32.1±0.4	32±0.3	32±0.3	32±0.2

Table S2: qPCR Primer sequences

Gene	Primer sequence(5′-3′)	Size(bp)	Reference
β-Actin	F:ATGACAGAGGATGAGGGTAGAAA<br>R:GAGAAGAGGTCCCGACGAATA	194	Designed by author
SODC	F：TGATACCAACCTGCCTCT<br>R：TGCGTGAACTTGAAATAAC	186	Designed by author
CATE	F：TGGAAACAATACGCCAATCT<br>R：AGTGATGAAATCCCAGAACAT	124	Designed by author
CYP3A	F：ATCGCCGCAGAAATCGTG<br>R：CGTAGCCAGCCAATAGAAAG	163	Designed by author
HSP90	F：GCTGTTTGCCGAAGTGAC<br>R：GAGGCTGAAGAATGGTATCT	144	Designed by author
HSP40	F：TGGTCAGATGCCAAGTCC<br>R：CGGCTAAATACAGGGTGC	96	Designed by author
CalM	F：ACCTCTTTCGTTTCGCTGAC<br>R：CTTCCTGAATCGCTGTTGCT	145	Designed by author
Casp8	F：GCACAGCCTACGACATTG<br>R：GACACGACCGTTCATTCA	108	Designed by author
Casp7	F：TTCAGTTCCCTTGCGTTTA<br>R：CCTCCCTTCCATCTACACC	175	Designed by author
ALG9	F：TCGTTATGGCTCCAAGAATC<br>R：ACCGTCGTGAGAACAGTTTC	193	Designed by author
Galaxin	F：CGATGCCACCCTTTCCC<br>R：CTGGTACGCCTACAGGTTTGT	137	Designed by author
GPX	F：GAATCATCTCGCCACCTGAC<br>R：GCATACTTCGGAGCCTACTGA	96	Designed by author
ATG5	ACTGTAAACCGACGCCTGAT<br>GGAACAAACTCCCGCAACA	173	Designed by author

Table S3: Data of various physiological indicators, the results are presented as means ± standard deviation. The results are presented as means ± standard deviation.

Variable				Zooxanthellae×10^6 (cell cm^{-2})				
		27℃		Mean±sd		30℃		Mean±sd
Coral	0.29	0.33	0.32	0.32±0.02	0.03	0.06	0.06	0.05±0.02
Coral exposed to algae	0.24	0.11	0.11	0.15±0.07	0.07	0.21	0.18	0.15±0.07
Coral contact with algae	0.15	0.22	0.36	0.24±0.11	0.12	0.28	0.28	0.23±0.09
				Chl *a* (μg cm^{-2})				
		27℃		Mean±sd		30℃		Mean±sd
Coral	5.3	4.1	6.7	5.34±1.3	0.25	0.47	0.27	0.33±0.12
Coral exposed to algae	1.8	1.8	1.1	1.54±0.4	0.29	3.47	3.35	2.37±1.8
Coral contact with algae	3.8	2.5	4.0	3.43±0.8	1.28	4.89	1.31	2.47±2.1
				Protein (mg cm^{-2})				
		27℃		Mean±sd		30℃		Mean±sd
Coral	1.17	0.72	0.59	0.83±0.3	0.33	0.31	0.48	0.37±0.09
Coral exposed to algae	0.54	0.71	0.29	0.52±0.21	0.38	0.64	0.75	0.59±0.19
Coral contact with algae	0.78	0.39	0.42	0.53±0.22	0.44	0.49	0.35	0.43±0.07
				Growth Rate (mg cm^{-2} d^{-1})				
		27℃		Mean±sd		30℃		Mean±sd
Coral	0.45	1.06	1.74	1.09±0.64	0.51	1.2	0.79	0.83±0.22
Coral exposed to algae	1.13	0.42	0.73	0.76±0.35	0.56	1.5	0.46	0.84±0.58
Coral contact with algae	0.97	0.77	0.53	0.76±0.22	0.41	0.49	0.61	0.5±0.1
				SOD (U mgprot^{-1})				
		27℃		Mean±sd		30℃		Mean±sd
Coral	149.1	231.1	241.6	207.3±50.6	426.9	635.4	341.3	467.8±151.3
Coral exposed to algae	316.8	282.3	515.3	371.5±125.8	213	288.4	227.7	243±40
Coral contact with algae	501.1	417.3	261.3	393.3±121.7	282.7	273.3	286.8	280.9±7
				CAT (U mgprot^{-1})				
		27℃		Mean±sd		30℃		Mean±sd
Coral	3.76	8.92	12.64	8.44±4.46	27.01	17.34	33.18	25.84±7.99
Coral exposed to algae	6.14	12.49	25.71	14.78±9.98	51.01	41.34	43.29	45.21±5.11
Coral contact with algae	48.54	53.79	51.73	51.35±2.64	14.46	24.4	25.03	21.3±5.93

Table S4: Two way ANOVA output of different variables for *A. hyacinthus* with bold values indicating significant effects on the variable. F=F-value; p=p-value(significance <0.05).

Variable	Source of variation	F	p
Growth Rate	Algae	F(2,8) = 0.7	0.4
	Temperature	F(1,4) = 0.7	0.5
	Interaction	F(2,8) = 0.3	0.7
Zooxanthellae	Algae	F(2,8) = 2.1	0.18
	Temperature	F(1,4) = 6.2	0.07
	Interaction	F(2,8) = 6.9	**0.02**
Chl *a*	Algae	F(2,8) = 1.4	0.3
	Temperature	F(1,4) = 5.1	0.09

	Interaction	F(2,8) = 10.8	**<0.01**
Protein	Algae	F(2,8) = 0.8	0.46
	Temperature	F(1,4) = 1.8	0.25
	Interaction	F(2,8) = 4.3	0.05
SOD	Algae	F(2,8) = 0.17	0.86
	Temperature	F(1,4) = 0.03	0.86
	Interaction	F(2,8) = 6.3	**0.02**
CAT	Algae	F(2,8) = 16.4	**<0.01**
	Temperature	F(1,4) = 2.8	0.17
	Interaction	F(2,8) = 43.5	**<0.01**

Table S5: Statistics summary of RNA-Seq of *A. hyacinthus*

Samples	Clean reads	Mapped reads	Comparison rate	Q30	GC content
LD	21 476 650	14 574 005	67.9%	96%	46.1%
LH	20 347 432	13 261 939	65.2%	94.6%	46.5%
LM	20 993 422	13 807 161	65.8%	94.4%	47.1%
HD	21 290 828	14 314 966	67.3%	95.9%	43.9%
HH	21 142 687	14 068 385	66.6%	95.9%	46.4%
HM	21 336 087	14 179 496	66.5%	96%	45.5%
Total	126 587 106				

Notes：Q30 represent 0.1% probabilities of base error，respectively

Table S6: DEGs statistics in all treatments. Select FDR < 0.001 and shown as LH, LM and HD groups vs LD group, HH and HM groups vs HD group.

DEGs	LH groups	LM groups	HD groups	HH groups	HM groups
Up-regulated	5 621	5 313	1 590	16	15
Down-regulated	6 413	7 160	11	2 861	313

Table S7: FPKM expression map of different biological processes regulated by DEGs. The results are presented as means ± standard deviation.

DEGs	LD groups	LH groups	LM groups	HD groups	HH groups	HM groups
Rab5	75.3±7.5	10.6±0.3	13.1±4.9	91.8±18.9	74.5±5.5	64.4±36.8
Rab10	0.5±0.1	42.7±6.8	32.7±6.5	0.6±0.5	0.5±0.2	16.9±28.6
Rab22	54±8.8	0.7±0.2	0.6±0.2	66.5±4.2	52.4±13.3	41.1±34.0
Rab11A	13.3±3.2	79.1±6.0	58.9±7.1	12.9±0.6	8.9±3.1	44.3±51.4
ATG5	0.2±0.1	12.5±2.4	10.7±2.7	0.4±0.1	0.2±0.1	3.8±6.0
ATG10	17.4±11.4	0.8±0.2	1.3±0.1	8.6±4.9	16.5±15.2	10.1±8.9
CalM	71.2±7.8	3.9±0.6	2.7±0.3	97.7±6.4	54.7±10.0	54.3±43.8

Galaxin	5.9±1.2	0.9±0.8	0.7±0.9	48.5±10.0	9.4±12.0	11.1±12.5
SODC	38.4±17.4	8.8±12.2	20.1±1.1	107.2±34.8	33.5±14.0	24.1±19.4
CAT	4.6±1.9	12.8±2.0	14.0±4.1	11.3±4.2	11.7±9.1	23.6±19.0
GPX	0.9±0.1	0.04±0.08	0.03±0.05	1.5±0.6	0.6±0.1	0.4±0.2
Hsp70	16.8±5.8	3.8±0.6	3.3±1.1	26.3±5.4	11.8±3.5	10.7±4.3
HSP40	7.2±0.72	27.2±8.4	20.8±4.9	11.2±1.0	7.5±1.2	14.6±10.1
CYP3A	9.5±1.9	0.1±0.1	0.2±0.2	20.9±3.1	12.3±2.5	7.6±6.6
TNFRSF14	3.1±3.0	0.7±0.1	0.4±0.2	8.7±5.3	2.8±3.4	1.4±0.8
TLR2	2.6±0.1	10.4±2.6	13.3±0.7	6.1±1.6	2.7±1.3	8.3±7.9
TRAF3	5.3±2.0	0.3±0.2	0.2±0.2	19.0±1.5	2.5±2.3	7.3±7.6
IFI47	3.2±0.2	3.3±0.1	4.4±0.9	15.1±9.5	3.4±0.5	5.5±1.2
TRAF5	2.9±2.9	0.9±1.0	0.8±0.8	9.1±7.1	0.9±0.2	5.4±7.7
MYD88	18.3±4.4	2.1±0.1	1.3±0.4	38.7±5.1	19.1±7.2	17.7±13.4
Casp3	0.3±0.1	10.0±0.8	9.7±0.6	1.2±0.6	0.3±0.1	5.4±8.2
Casp8	20.7±1.9	3.0±1.0	0.9±1.5	25.6±1.3	18.9±5.7	15.9±11.3
Casp7	4.3±1.0	11.0±3.0	5.0±3.3	9.9±3.5	4.4±1.6	9.1±5.7
FADD	7.8±1.7	5.0±0.7	4.7±0.7	11.5±0.7	7.0±1.5	7.1±1.5
HIRA2	1.1±0.4	9.9±2.3	8.1±1.6	1.6±0.2	0.75±0.3	4.9±6.7

Table S8: The log_2FoldChange value of qRT-PCR and RNA-seq. Compared with LD group, DEGs verification of LH, LM and HD groups . And compared with HD group, the DEGs verification of HH and HM. The results are presented as means ± standard deviation.

Group	DEGs	qRT-PCR	RNA-seq
LH	Casp8	-1.4±1.2	-2.9
	Casp7	1.1±0.6	1.3
	CYP3A	-2.0±2.9	-3.3
	ATG5	3.1±0.5	5.9
LM	Casp8	-1.6±0.4	-6.3
	Hsp90	-1.1±0.3	-1.3
	ATG5	3.2±0.4	5.5
HD	CAT	0.6±0.6	2.3
	SODC	2.3±0.7	2.5
	CYP3A	1.3±0.9	2.1
	CalM	1.2±0.7	1.9
	Galaxin	5±0.8	3.9
	Casp7	3.5±0.4	2.2
HH	SODC	-2.9±0.4	-3.0
	CYP3A	-1.8±0.2	-2.0
	Hsp40	-1.6±1.1	-2.0
	CalM	-1.6±0.7	-2.1
	CASP7	-3.2±1.2	-2.7
HM	ALG9	-1.3±0.4	-3.0
	CalM	-1.6±0.7	-2.1
	GPX	-1.6±2.4	-2.8

Table. S9: Distribution of gene ontologies which were signifificantly enriched in KEGG pathways of LH , LM, HD, HH and HM treatments. The difference gourps selected as LH, LM and HD compared with LD, HH and HM compared with HD.

Group	Pathway	ko ID	DEGs in Pathway	All gene in Pathway	P-value
LH	FoxO signaling pathway	ko04068	102	193	<0.01
	Inositol phosphate metabolism	ko00562	71	140	<0.01
	mTOR signaling pathway	ko04150	139	311	<0.01
	Endocytosis	ko04144	210	491	<0.01
	Autophagy - animal	ko04140	164	375	<0.01
	Apoptosis - multiple species	ko04215	39	72	<0.01
	Various types of N-glycan biosynthesis	ko00513	48	96	<0.01
	Glycosphingolipid biosynthesis - lacto and neolacto series	ko00601	14	21	<0.01
	Glycosaminoglycan biosynthesis - keratan sulfate	ko00533	7	8	<0.01
	Neuroactive ligand-receptor interaction	ko04080	103	233	<0.01
	Other glycan degradation	ko00511	27	50	<0.01
	beta-Alanine metabolism	ko00410	42	85	<0.01
	Glycosaminoglycan degradation	ko00531	33	64	<0.01
	Cell adhesion molecules	ko04514	14	22	<0.01
	TGF-beta signaling pathway	ko04350	50	105	<0.01
	Other types of O-glycan biosynthesis	ko00514	28	54	0.01
	Lysine degradation	ko00310	64	142	0.01
	Notch signaling pathway	ko04330	54	118	0.02
	Mannose type O-glycan biosynthesis	ko00515	15	27	0.03
	Wnt signaling pathway	ko04310	115	278	0.03
LM	mTOR signaling pathway	ko04150	151	311	<0.01
	Autophagy - animal	ko04140	176	375	<0.01
	Glycosaminoglycan degradation	ko00531	39	64	<0.01
	FoxO signaling pathway	ko04068	97	193	<0.01
	TGF-beta signaling pathway	ko04350	55	105	<0.01
	Inositol phosphate metabolism	ko00562	69	140	<0.01
	Endocytosis	ko04144	213	491	<0.01
	Neuroactive ligand-receptor interaction	ko04080	107	233	<0.01
	Glycosaminoglycan biosynthesis - keratan sulfate	ko00533	7	8	<0.01
	Apoptosis - multiple species	ko04215	38	72	<0.01
	Glycosphingolipid biosynthesis - lacto and neolacto series	ko00601	14	21	<0.01
	Various types of N-glycan	ko00513	48	96	<0.01

	biosynthesis				
	Other types of O-glycan biosynthesis	ko00514	29	54	0.01
	Cell adhesion molecules	ko04514	14	22	0.01
	ECM-receptor interaction	ko04512	67	144	0.02
	Other glycan degradation	ko00511	26	50	0.03
	Glycosaminoglycan biosynthesis - heparan sulfate / heparin	ko00534	19	35	0.03
	Wnt signaling pathway	ko04310	119	278	0.04
	Mannose type O-glycan biosynthesis	ko00515	15	27	0.04
	Lysosome	ko04142	182	440	0.05
HD	ErbB signaling pathway	ko04012	7	19	<0.01
	Cytosolic DNA-sensing pathway	ko04623	5	20	<0.01
	TNF signaling pathway	ko04668	5	22	<0.01
	Cell adhesion molecules	ko04514	5	22	<0.01
	Herpes simplex virus 1 infection	ko05168	11	107	0.01
	Toll-like receptor signaling pathway	ko04620	5	36	0.02
	Inositol phosphate metabolism	ko00562	12	140	0.03
	MAPK signaling pathway	ko04010	9	95	0.03
	FoxO signaling pathway	ko04068	15	193	0.04
	Phenylalanine metabolism	ko00360	5	41	0.04
	Glyoxylate and dicarboxylate metabolism	ko00630	10	115	0.04
	Tryptophan metabolism	ko00380	10	121	>0.05
	Glycosaminoglycan degradation	ko00531	6	64	>0.05
	Phosphatidylinositol signaling system	ko04070	16	238	>0.05
	Ubiquitin mediated proteolysis	ko04120	23	367	>0.05
	Ether lipid metabolism	ko00565	5	52	>0.05
	Butanoate metabolism	ko00650	5	54	>0.05
	N-Glycan biosynthesis	ko00510	6	70	>0.05
	Autophagy - animal	ko04140	23	375	>0.05
	Protein processing in endoplasmic reticulum	ko04141	28	472	>0.05
HH	Endocytosis	ko04144	67	491	<0.01
	TNF signaling pathway	ko04668	7	22	<0.01
	NOD-like receptor signaling pathway	ko04621	17	93	<0.01
	Apoptosis - multiple species	ko04215	14	72	<0.01
	ErbB signaling pathway	ko04012	6	19	<0.01
	Vitamin digestion and absorption	ko04977	3	5	<0.01
	Cell adhesion molecules	ko04514	6	22	0.01
	Lysosome	ko04142	53	440	0.01
	Ether lipid metabolism	ko00565	10	52	0.01
	Sulfur relay system	ko04122	4	13	0.02

	Glycosaminoglycan biosynthesis - heparan sulfate / heparin	ko00534	7	35	0.03
	Basal transcription factors	ko03022	10	59	0.03
	Glycerophospholipid metabolism	ko00564	20	151	0.04
	ECM-receptor interaction	ko04512	19	144	0.049
	Phosphonate and phosphinate metabolism	ko00440	4	18	>0.05
	MAPK signaling pathway	ko04010	13	95	>0.05
	FoxO signaling pathway	ko04068	23	193	>0.05
	N-Glycan biosynthesis	ko00510	10	70	>0.05
	Amino sugar and nucleotide sugar metabolism	ko00520	15	117	>0.05
	Toll-like receptor signaling pathway	ko04620	6	36	>0.05
HM	Neuroactive ligand-receptor interaction	ko04080	5	233	0.02
	Cytokine-cytokine receptor interaction	ko04060	1	8	0.049
	Arachidonic acid metabolism	ko00590	3	140	>0.05
	Apoptosis - multiple species	ko04215	2	72	>0.05
	Glutathione metabolism	ko00480	3	182	>0.05
	Herpes simplex virus 1 infection	ko05168	2	107	>0.05
	Phenylalanine, tyrosine and tryptophan biosynthesis	ko00400	1	26	>0.05
	Vitamin B6 metabolism	ko00750	1	26	>0.05
	Taurine and hypotaurine metabolism	ko00430	1	27	>0.05
	Pyrimidine metabolism	ko00240	2	114	>0.05
	Influenza A	ko05164	1	28	>0.05
	Ubiquinone and other terpenoid-quinone biosynthesis	ko00130	1	28	>0.05
	Notch signaling pathway	ko04330	2	118	>0.05
	Peroxisome	ko04146	3	263	>0.05
	Phenylalanine metabolism	ko00360	1	41	>0.05
	ECM-receptor interaction	ko04512	2	144	>0.05
	Nucleotide excision repair	ko03420	2	145	>0.05
	Riboflavin metabolism	ko00740	1	42	>0.05
	Purine metabolism	ko00230	3	278	>0.05
	Thiamine metabolism	ko00730	1	49	>0.05
